# Ischemia/Reperfusion Injury following Acute Myocardial Infarction: A Critical Issue for Clinicians and Forensic Pathologists

**DOI:** 10.1155/2017/7018393

**Published:** 2017-02-13

**Authors:** Margherita Neri, Irene Riezzo, Natascha Pascale, Cristoforo Pomara, Emanuela Turillazzi

**Affiliations:** ^1^Section of Forensic Pathology, Morphology, Surgery and Experimental Medicine Department, University of Ferrara, Ospedale “Sant'Anna”, Via Fossato di Mortara 70, 44121 Ferrara, Italy; ^2^Section of Forensic Pathology, Clinical and Experimental Medicine Department, University of Foggia, Ospedale Colonnello D'Avanzo, Viale Degli Aviatori 1, 71100 Foggia, Italy

## Abstract

Acute myocardial infarction (AMI) is a leading cause of morbidity and mortality. Reperfusion strategies are the current standard therapy for AMI. However, they may result in paradoxical cardiomyocyte dysfunction, known as ischemic reperfusion injury (IRI). Different forms of IRI are recognized, of which only the first two are reversible: reperfusion-induced arrhythmias, myocardial stunning, microvascular obstruction, and lethal myocardial reperfusion injury. Sudden death is the most common pattern for ischemia-induced lethal ventricular arrhythmias during AMI. The exact mechanisms of IRI are not fully known. Molecular, cellular, and tissue alterations such as cell death, inflammation, neurohumoral activation, and oxidative stress are considered to be of paramount importance in IRI. However, comprehension of the exact pathophysiological mechanisms remains a challenge for clinicians. Furthermore, myocardial IRI is a critical issue also for forensic pathologists since sudden death may occur despite timely reperfusion following AMI, that is one of the most frequently litigated areas of cardiology practice. In this paper we explore the literature regarding the pathophysiology of myocardial IRI, focusing on the possible role of the calpain system, oxidative-nitrosative stress, and matrix metalloproteinases and aiming to foster knowledge of IRI pathophysiology also in terms of medicolegal understanding of sudden deaths following AMI.

## 1. Introduction

Acute myocardial infarction (AMI) is a leading cause of morbidity and mortality in the world [1]. Reperfusion strategies are the current standard therapy for AMI [2, 3]. They may, however, result in paradoxical cardiomyocyte dysfunction and worsen tissue damage, in a process known as “reperfusion injury” [4–9]. Ischemic reperfusion injury (IRI) typically arises in patients presenting with an acute ST-segment elevation myocardial infarction (STEMI), in whom the most effective therapeutic intervention is timely and effective myocardial reperfusion [7, 10–14]. Reperfusion itself is known as a “double-edged sword” [4, 15] due to the spectrum of reperfusion-associated pathologies. Outcomes subsequent to IRI accrue in a time-dependent fashion [16], beginning with oxidative stress, inflammation, intracellular Ca^2+^ overload, and rapidly proceeding to irreversible cell death by apoptosis and necrosis [13, 16]. Different forms of myocardial IRI are recognized, of which only the first two are reversible: reperfusion-induced arrhythmias, myocardial stunning, microvascular obstruction, and lethal myocardial reperfusion injury [13].

In particular, sudden death is the most common pattern for ischemia-induced lethal ventricular arrhythmias (VAs) during the acute phase of myocardial infarction [17], and it is well known that reperfusion itself can lead to life-threatening VAs [17] and, ultimately, induce sudden mortality.

The exact mechanisms of IRI are not fully known [18]. Molecular, cellular, and tissue alterations such as cell death, inflammation, neurohumoral activation, and oxidative stress are considered to be of paramount importance for IRI development [10, 19]. However, comprehension of the exact pathophysiological mechanisms of IRI [20, 21] remains a challenge for clinicians [22, 23], and the existence of reperfusion injury is still a matter of debate in the scientific community, essentially due to a lack of a definitive clinical documentation. Many gaps still exist between experimental animal models and human clinical experience, with subsequent difficulties in translating experimental results on cardioprotection to clinical practice [22–24]. Despite the difficulties that still exist in fully comprehending myocardial IRI, early and aggressive reperfusion strategies remain the most important intervention and are strongly advocated. The development of ischemic conditioning strategies to limit the extent of infarcted tissue caused by ischemia/reperfusion injury markedly enhances the ability of the heart to withstand an ischemic insult [25].

Finally, myocardial IRI is a critical issue also for forensic pathologists since sudden death may occur despite timely reperfusion following AMI, that is one of the most frequently litigated areas of cardiology practice [26, 27].

In this paper we explore the literature regarding the pathophysiology of myocardial IRI, focusing on the possible role of the calpain system, oxidative-nitrosative stress, and matrix metalloproteinases. We discuss these mechanisms within the broad scenario of IRI, also discussing the medicolegal issues related to sudden deaths occurring during the acute phase of myocardial infarct following reperfusion interventions.

## 2. The Calpain System

The process of IRI is not yet completely understood in its underlying pathophysiological mechanisms. Several pathways have been proposed, including cytosolic and mitochondrial Ca^2+^ overload, release of reactive oxygen species (ROS), acute inflammatory response, and impaired metabolism [20, 21]. These alterations may collaboratively act and produce irreversible damage to ischemic reperfused cardiomyocytes.

The possibility that the calpain system could play a role in generating myocardial IRI has been experimentally investigated in the literature [28–32], and several studies have focused on the effects of calpain inhibitors in improving myocardial dysfunction in different animal models [33–37]. Calpains are a family of Ca^2+^-dependent nonlysosomal cystein proteinase localized in the cytosol in their inactive form [38]. Calpain activation, which may occur under several conditions, is thought to be a key mechanism in activating a number of substrates such as growth factor receptors, cytoskeletal proteins, microtubules associated proteins, and mitochondria, so playing a crucial role in cell cycle, apoptosis, and differentiation [38–40].

The calpain superfamily is complex, and more than 25 calpains or calpain-like molecules have been discovered. Calpains 1 and 2 are biologically activated when they arrange as dimer with a 30 kDa subunit. Both biologically active calpains are usually called *μ*-calpain (calpain 1 + 30 kDa subunit) and m-calpain (calpain 2 + 30-kDa subunit). The terms *μ*-calpain and m-calpain indicate, respectively, the micromolar and millimolar Ca^2+^ concentrations required for their activation [19]. Calpains may appear in the form of both “ubiquitous” isoenzymes that are present in almost all cells (such as *μ*-calpain, m-calpain, and calpains 5, 7, 10, 13, and 15) and “tissue specific” calpains expressed only in special tissues and cells, such as calpains 3 and 6 and others [31].

In brief, it has been hypothesized that, under physiological conditions, inactive calpains are stored in cellular cytosol and bound in a substrate competitive manner to their endogenous inhibitor calpastatin. The elevation of intracellular calcium levels is the key to the calpain activation process. Calpain conformational changes permit its translocation into cellular membrane, where phospholipids reduce the Ca^2+^ threshold for calpain activation or close the Ca^2+^ channels leading up to protein activation [41]. Several pathological cardiac events are associated with an imbalance of calcium homeostasis related to myocardial ischemia/reperfusion injury [29–31]. Experimental studies on isolated perfused mammalian hearts demonstrated an increase in intracellular Ca^2+^ concentrations in response to ischemia/reperfusion [31, 41]. Myocardial ischemia favours intracellular ion accumulation (sodium, calcium) till dropping in pH and tissue acidosis. Reperfusion evokes rapid alterations in ion flux and interacts with ischemia in altering the physiology of ion exchange [42]. Among others, a final result of the dangerous interplay between ischemia and reperfusion is intracellular calcium overload.

The kinetics of calpain activation are not completely understood, and whether or not translocation to the sarcolemma is needed for calpain activation during IRI remains undetermined [43]. In their elegant experiment, Hernando et al. [37] suggested that calpain translocation to the cardiomyocytes membranes during ischemia is independent of its activation since intracellular acidosis occurring during ischemia is likely to inhibit calpain activation. As intracellular pH normalizes following reperfusion, calpain activation occurs. Despite translocation, calpain seems to remain inactive even after 60 minutes of ischemia and only on reperfusion is it activated [37].

Activated calpain has a number of substrates such as growth factor receptors, cytoskeletal proteins, microtubules associated proteins, and mitochondria, thus playing a crucial role in the processes of cell cycle, apoptosis, and differentiation, negatively affecting cardiomyocyte function.

Firstly, the calpain system is part of the integrated proteolytic system which is crucial to the maintenance of the structure and function of the cardiac sarcomere. An imbalance of this system is the key to the sarcomeric dysfunction linked to several cardiovascular diseases, including hypoxia, IRI, myocardial infarction, and end-stage heart failure. Protein degradation (proteolysis) within cardiac sarcomere is regulated mainly by three systems: the ubiquitin proteasome system (UPS); autophagy/lysosomal degradation; and the calpain system [44]. Degradation of myofibrillar proteins involved in the contractile process is an effect of calpain activation. The degradation process following IRI involves either structural or regulatory proteins of contractile apparatus. In vitro study [45] showed that many of these proteins are potential targets of activated calpains, thus contributing to the development of postischemic injury in the human myocardium. Several experimental studies demonstrated that the loss/disorganization of T-tubules structure is a key factor in heart failure development [46–48]. Calpain-mediated disruption of T-tubules integrity through the proteolysis of junctophilin is demonstrated to be one of the major factors involved in an experimental model of cardiac muscle failure [49, 50].

Calpain deregulation is known to be an effective mechanism of apoptosis induction in cardiac sarcomeres through different pathways [51–53], and apoptosis of myocardial cells is considered an important mechanism of IRI [54–56].

**Figure 1:**
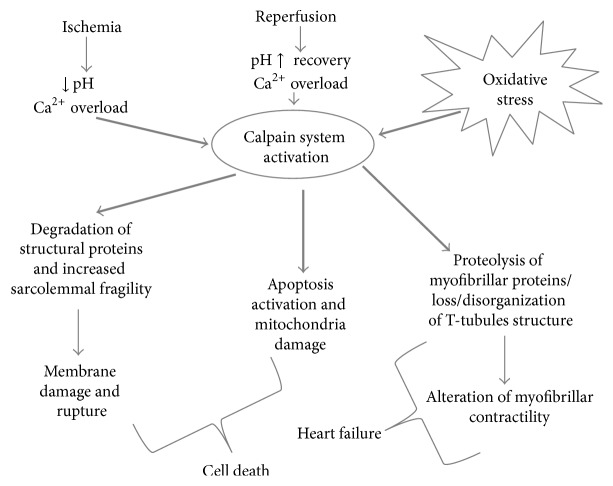
Schematic representation of calpain activation during myocardial IRI. Ca^2+^ overload and pH recovery in reperfusion phase are crucial in the activation of the calpain system. Increased sarcolemmal fragility may lead to membrane rupture and cell death. In addition, both the death-receptor and mitochondrial mediated apoptotic pathways seem to be affected by calpain activation. The degradation of myofibrillar proteins and the loss/disorganization of T-tubules structure are key factors in post-MI heart failure development.

Conclusively, an uncontrolled activation of calpain has been found to be implicated in the pathophysiology of several cardiovascular disorders [57] including myocardial IRI [58], and the inhibition of calpains has been shown to attenuate myocardial stunning and reduce infarct size after ischemia reperfusion [59] (Figure 1). However, the exact role of calpain in acute myocardial IRI remains controversial [60].

## 3. Oxidative Stress and Mitochondria

An oxidant and antioxidant imbalance (oxidative stress) favours the accumulation of oxidants, from both increased ROS production and decreased ROS scavenging ability, thus leading to cellular damage in the cardiomyocytes [61]. Oxidative stress is often associated with elevated levels of ROS or reactive nitrogen species (RNS) in the cellular and subcellular levels [61], leading to proteins, lipids, and DNA damage [62]. Furthermore, in cardiomyocytes, increased ROS/RNS levels can induce alterations of proteins involved in excitation-contraction coupling with increased susceptibility to proteolysis [62–65].

In the first few minutes IRI, and especially myocardial reperfusion, induces a high production of ROS by a variety of sources [66–69]. Since Arroyo et al. provided direct evidence of ROS formation during myocardial ischemia and postischemic reperfusion by trapping these free radicals using nitrone DMPO [70], several preclinical and clinical studies [71–74] have demonstrated the potential cardioprotective value of antioxidants. While small amounts of ROS could result in cardioprotection via preconditioning [75], the excessive production of ROS during reperfusion seems especially important in inducing injury.

Mechanisms leading up to the dysfunction and the initial sources of ROS during IRI are not completely clear [76]. Nitric oxide (NO) production is considered a key factor in IRI. NO is an important bioactive substance which plays an important role in the regulation of normal body function and disease occurrence, and it is recognized as an ubiquitous signalling molecule with a multitude of biological actions and targets. Signalling may involve direct reactions between NO and a molecular target or can occur through indirect reactions of secondary ROS [77]. In fact, actions of NO are multifaceted, and its interactions with oxygen or oxygen-related reactive intermediates (e.g., superoxide) yield numerous RNS and ROS. These account for most of the so-called indirect effects attributed to NO through oxidation, nitrosation, and nitrate reactions referred to as oxidative, nitrosative, and nitrative stress, respectively. The physiological production of NO in the heart maintains coronary vasodilator tone and inhibits platelet aggregation and neutrophil and platelet adhesion, so performing an active role in cardioprotection [78–80]. Beyond its beneficial effects, it has been speculated that NO excess can induce cellular injury either due to direct toxicity [81, 82] and to the reaction with superoxide (O2^−^) to form the potent oxidant peroxynitrite (ONOO2) [83] which in turn exerts cytotoxicity via its reaction with a variety of molecular targets [84, 85]. The formation of highly reactive species, such as peroxynitrite, is a possible mechanism by which NO elicits its dangerous effects [83].

Much about NO biological actions remains contradictory, especially with regard to pathophysiologic disturbances in NO signalling. There is an ongoing debate about the levels of NO involved and whether there is a clearly defined threshold at which NO shifts from being beneficial to being destructive. Some authors hypothesize that the biological function of NO depends mostly on concentration and time course of exposure to NO, supposing that cytotoxic events, such as arrest of the cell cycle, cell senescence, or apoptosis, can occur at high NO concentrations [86]. However, other authors suggest that the chemical and biological reactivity of NO that has been studied using very high NO concentrations is of doubtful physiological relevance [87].

Zhang and Cai have shown that exogenously applied netrin-1 exerts robust cardioprotective effects against IRI, via an increase in NO formation [88]; the same group have further demonstrated that endogenously increased NO production could mediate cardioprotection by modulating oxidative stress and mitochondrial function [76]. Under physiological oxidative stress, NO mediates S-nitrosylation of critical protein thiols and thus averts them from further oxidative modifications by ROS, thereby rendering cardioprotection [89]. It is argued that NO protects the heart against IRI [79, 90]; however, excessive NO formation is thought to contribute to contractile dysfunction [91, 92].

During reperfusion NO release may be stimulated through a number of mechanisms including the change in shear stress in the coronary vasculature during reperfusion, increased intracellular Ca^2+^ levels as a result of ischemia, and the thermodynamically favoured production of NO from L-arginine and molecular oxygen due to reperfusion [93, 94]. NO is produced endogenously within the myocardium by three distinct isoforms of NO synthase (NOS) [95]. Neuronal NOS (NOS1) and endothelial NOS (NOS3) are constitutively expressed within cardiomyocytes while inducible NOS (NOS2) is only expressed within cardiomyocytes during inflammatory responses which occur during many pathophysiological conditions of the myocardium [96].

**Figure 2:**
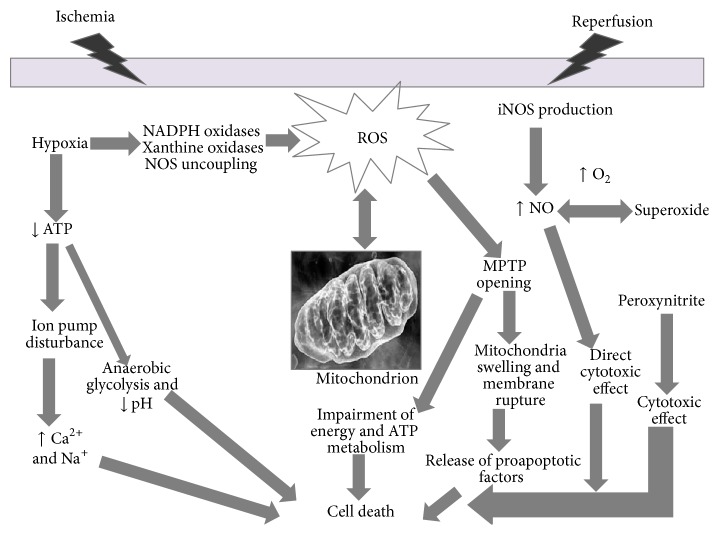
Schematic representation of oxidative stress contributing to tissue injury and cell death in IRI. Following ischemia, hypoxia results in reduction of ATP production, ion pump function unbalance, leading to overload of Na^+^ and Ca^2+^, activation of anaerobic glycolysis, and, finally, reduction of pH. During the initial ischemic phase, the activation and upregulation of enzymes (such as NADPH oxidase, a superoxide-generating enzyme comprising a membrane-bound catalytic subunit) occurs, that are capable of producing ROS, when molecular oxygen is reintroduced in the reperfusion phase. ROS induces cell dysfunction and death via other mechanisms: activation of metalloproteinases and calpains, mitochondrial permeability transition pore (MPTP) opening which contributes to swelling and lysis of cells. This may elicit the release of proapoptotic factors in the cytosol, thus contributing to cell death. ROS indirectly interact with nitric oxide (NO) production, partly mediated by the inducible NOS (iNOS), the high-capacity NO-producing enzyme. Unlike the other two NOS isoforms, iNOS is not constitutively expressed in cells, and its production is elicited by several stimuli like IRI. NO cytotoxic effects are both direct and indirect mediated by NO reaction with superoxide to form the potent oxidant peroxynitrite which in turn exerts cytotoxicity.

**Figure 3:**
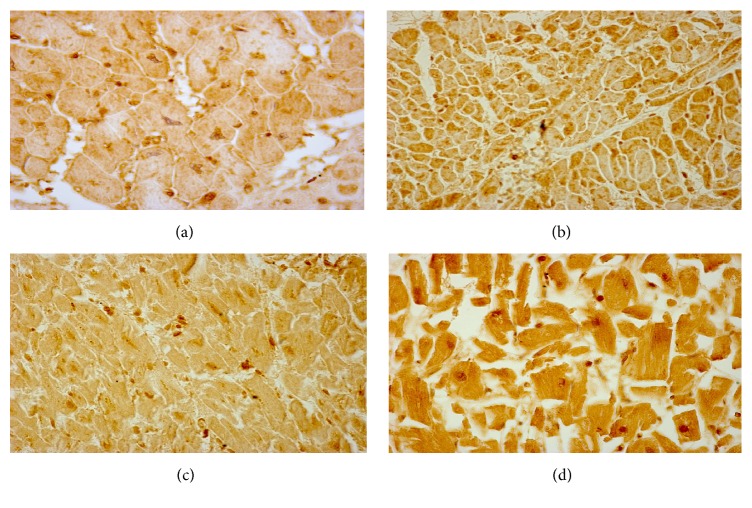
Histomorphological pictures showing the phenotypic results of altered pathways in IRI. (a) Mild calpain 1 expression in the left ventricle cardiac tissue of a patient who died following early reperfused AMI (calpain 1, antibody anti-calpain 1, Santa Cruz, USA). (b) NOX2 expression in the left ventricle cardiac tissue of a patient who died following prompt fibrinolysis in acute STEMI. (c) Strong immunopositivity to anti-nitrotyrosine antibody (Abcam, Cambridge, UK). (d) Mild immunopositivity to anti-iNOS (inducible nitric oxide synthase) antibody (Santa Cruz, CA, USA) in the left ventricle sample of a patient who died following reperfusion therapy in STEMI.

Mitochondria play a critical role in the pathogenesis of myocardial IRI. They occupy 30–50% of the cardiomyocyte cytoplasmic volume and are critical in cardiac energy balance since energy supply for cardiomyocytes is mostly derived from mitochondrial oxidative phosphorylation (OXPHOS). On the other hand, they are a favoured target of intracellular damage [97–99]. These cell organelles are the major contributors of ROS as well as the major target for ROS-caused damage [100–106]. Mitochondrial dysfunction, reflected in the structure, function, and number of mitochondria within the cardiomyocyte, leads to diminished energy production, loss of myocyte contractility, altered electrical properties, and eventual cardiomyocyte cell death [100]. In this context, the mitochondrial permeability transition pore (MPTP) is thought to play a critical role in myocardial IRI (Figures 2 and 3)

MPTP refers to a mitochondrial channel which mediates the abrupt change, or transition, in inner mitochondrial membrane permeability which occurs under certain conditions [107]. The opening of the MPTP renders the inner mitochondrial membrane nonselectively permeable to molecules less than 1.5 kDa and elicits mitochondrial membrane depolarization and uncoupling of oxidative phosphorylation. It also favours collapsing the mitochondrial membrane potential, and uncoupling oxidative phosphorylation, thus leading to impairment of energy and ATP metabolism and cell necrosis [108–111]. MPTP opening also causes mitochondrial swelling, and outer mitochondrial membrane rupture, thus favouring the deposition of proapoptotic factors such as cytochrome *c* and SMAC/Diablo from the intermembranous space into the cytosol, thereby initiating apoptotic cell death [107].

During ischemia/reperfusion, intertwined biochemical events occur leading to MPTP opening. In the ischemic period, following factors such as Ca^2+^, long-chain fatty acids, and ROS accumulation, the likelihood that MPTP will occur upon reperfusion gradually increases [112, 113]. During ischemia, due to increased glycolysis, an accumulation of lactic acid and reduction of pH occur. To restore the pH, the Na^+^/H^+^ antiporter is activated, but it acts inefficiently because Na^+^ cannot be pumped out of the cell, as the Na^+^/K^+^ ATPase is inhibited by the absence of intracellular ATP. Consequently, the cytosolic Ca^2+^ concentration increases. Moreover, the existing decrease in the adenine nucleotide concentration, which is associated with an increased phosphate concentration, is likely to sensitize MPTP opening in response to Ca^2+^; however, low pH inhibits the opening. When reperfusion occurs, the mitochondria recover their ability to respire and rescue the sustained mitochondrial membrane potential, which is required for ATP synthesis. In addition, strong production of ROS occurs when the inhibited respiratory chain is reexposed to oxygen. Thus, the following resulting conditions are nearly optimal for MPTP opening: high Ca^2+^ levels within the mitochondrial matrix, increased levels of phosphate and oxidative stress, depletion of adenine nucleotide concentration, and rapid restoration of physiological value of pH [113–115].

In their milestone paper, Griffiths and Halestrap [116] demonstrated that MPTP are closed during ischemia and open the first few (2-3) minutes of reperfusion. Subsequent data has confirmed that pore opening occurs during reperfusion of the heart after ischemia, but not in the ischemic period [107]. Thus MPTP is an important new target for cardioprotection during reperfusion [114].

## 4. The Matrix Metalloproteinases

One group of enzymes that is important in mediating IRI injury is the family of matrix metalloproteinases (MMPs). The MMPs are a large family of calcium-dependent, zinc-containing endopeptidases that have the ability to remodel the extracellular matrix in both physiological and pathological processes. MMPs are regulated at different levels including transcriptional, posttranscriptional, and posttranslational levels. Moreover, they are controlled via their endogenous inhibitors, the tissue inhibitor of metalloproteinases (TIMPs), and by their intra- and extracellular localization [117]. Of all MMPs, MMP-2 (also known as gelatinase A or type IV collagenase) plays a critical role in cardiovascular diseases [117]. MMP-2 activity is also regulated via nonproteolytic posttranslational modifications of the full-length zymogen form, by *S*-glutathiolation, *S*-nitrosylation, and phosphorylation [118–120]. The NO product, ONOO^−^, directly activates MMP-s 2 [118] via a nonproteolytic mechanism involving the *S*-glutathiolation of the propeptide cysteine sulfhydryl group in a reaction requiring only micromolar concentrations of ONOO^−^ in conjunction with normal intracellular levels of glutathione [119]. In turn, it was demonstrated that ONOO^−^ inactivate TIMP-4 and TIMP-1, leading to a net increase in MMP activity [118].

In IRI, the sudden availability of molecular oxygen during reperfusion reenergizes mitochondria and reactivates the electron transport chain, causing a significant increase in the biosynthesis of ROS (including ONOO^−^) [94, 121] which stimulates MMP-2 activity [118].

It has been demonstrated that MMP-2 exerts rapid effects in modulating different cellular functions independent of its action on the extracellular matrix (ECM). These include effects on platelet aggregation [122], vascular tone [123, 124], and acute mechanical dysfunction of the heart immediately after ischemia and reperfusion [125, 126]. In ischemia/reperfusion, injury may result in the partial proteolysis of the thin-filament regulatory protein troponin I (TnI) [60, 124, 125, 127–129], and studies on animal models have validated this observation, showing that MMP-2 degrade Tn I myofilaments [130].

MMP-2 has a proapoptotic role as demonstrated in adult rat cardiomyocyte by Menon et al. who show that inhibition of MMP-2 inhibits *β*-AR-stimulated apoptosis [131, 132]. Furthermore, MMP-2 is present in mitochondria [130], and cardiac-specific transgenic expression of active MMP-2 causes abnormalities in mitochondria ultrastructure, impaired respiration, increased lipid peroxidation, cell necrosis, and reduced recovery of contractile performance during post-IRI [133].

Finally, a complex interplay exists between the calpain and MMP systems since there appears to be overlap in the substrates and/or biological actions of MMP-2 and calpains in various cellular pathways [117]. Kandasamy et al. have hypothesized that either MMP-2 targets a subset of proteins similar to calpain, or calpain has been incorrectly identified as the protease responsible for some intracellular proteolytic activities. Indeed, much of the evidence for calpain degradation of substrates in cardiac cells rests on the use of calpain inhibitors such as calpastatin, which has been found to inhibit MMP-2 activity in vitro [117].

Other MMPs are thought to be involved in myocardial injury following AMI, such as MMP-9, first known as 92-kDa type IV collagenase or gelatinase B, a structurally complex metalloproteinase that intervenes in the degradation of ECM in a large spectrum of physiology and pathophysiology processes involving tissue remodelling, including cardiac remodelling after AMI.

MMP-9 is expressed in the heart by endogenous cardiac cell types (e.g., cardiomyocytes, endothelial cells, and fibroblasts) and is also produced by nonresident cells that infiltrate the infarct in response to ischemic injury (e.g., leukocytes) [134–136].

Different and opposite functions have been hypothesized for MMP-9. Potential detrimental consequences of MMP-9 release and activation may include stimulating inappropriate extracellular matrix degradation, activating inflammatory mediators, and/or increasing capillary permeability [137, 138]. On the other hand, potential beneficial effects of early MMP-9 activation include removing matrix and necrotic myocytes, releasing growth factors and cell surface receptors, remodelling the extracellular matrix for scar formation, processing inflammatory mediators such as interleukin-1*β*, and influencing angiogenesis [137]. MMP-9 has been correlated with an increase in infarct size and left ventricle fibrosis after experimental AMI [138–142].

Furthermore, increased myocardial MMP-9 expression or activity has been found in experimental myocardial injuries such as permanent coronary artery occlusion [143, 144] or reperfusion injury model in animals [145, 146], and the possible role of MMP-9 activation in myocardial IRI has been explored [147].

**Figure 4:**
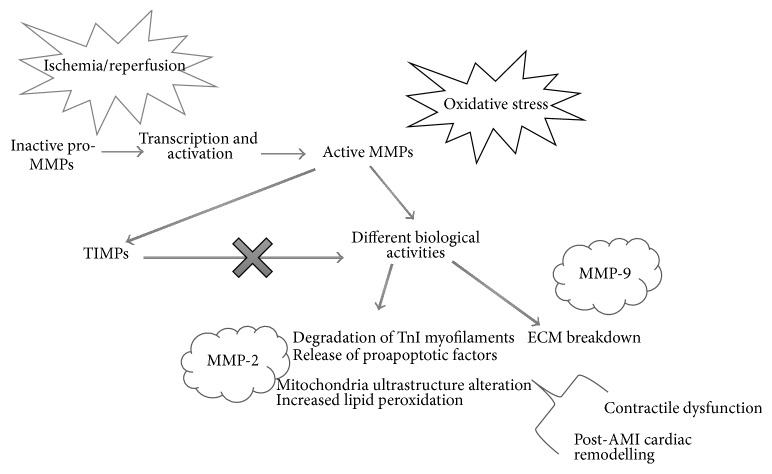
The matrix metalloproteinases system. MMPs activity results from different levels of regulation: transcription, activation, and inhibition by tissue inhibitors of metalloproteinases (TIMPs). During ischemia/reperfusion, oxidative stress stimulates the activity of MMPs, like MMP-2. Several biological activities of MMPs may contribute to myocardial contractile dysfunction and cell death. MMPs can both degrade extracellular matrix (ECM) and modulate different cellular mechanisms, thus leading to contractile dysfunction and modulation of cardiac remodelling and healing.

Following myocardial acute ischemia and reperfusion, neutrophil-derived MMP-9 is released in the myocardium and its levels increase as early as several minutes after AMI, remaining high for the first week in many animal models [137, 144, 148]. In the early phase of reperfusion, MMP-9 activation is likely to be localized in the perineutrophil area and might be initiated by neutrophils adhering to the ECM [137]; its temporal trend mirrors leukocyte infiltration [149]. The action of MMP-9 appears to be complex; it directly degrades ECM proteins and activates cytokines and chemokines to regulate tissue remodelling. MMP-9 deletion or inhibition has proven to be beneficial in a variety of animal models of cardiovascular disease. On the other hand, MMP-9 cell-specific overexpression has also proven beneficial [137, 144, 146, 150] (Figure 4).

## 5. Ischemia/Reperfusion Injury and Medicolegal Issues

Early reperfusion reduced mortality in AMI so that, in many countries, the hospital mortality has declined to about 5% [151]. There is no doubt that early reperfusion, both pharmacological and mechanical, is the only way to prevent progression to myocardial necrosis and thus to limit the size of the infarct. However, myocardial IRI has been described following reperfusion therapies including percutaneous coronary intervention (PCI), thrombolysis, and coronary bypass grafting [7, 13, 16].

VAs upon reperfusion have been recognized since the advent of recanalization techniques [152]; however their pathophysiological and prognostic significance is still controversial [153]. Several arrhythmogenic mechanisms have been proposed to be involved in IRI-induced arrhythmias [154, 155]. Some of VAs that occur almost directly at the moment of reperfusion (namely, ventricular premature beats and accelerated idioventricular rhythms) are usually harmless and well tolerated [152]; however it has been reported that ventricular tachycardia and ventricular fibrillation occurring immediately after reperfusion remain the most important causes of sudden death following restoration of blood flow [156, 157].

Severe arrhythmias may not be common but the fact that they are life threatening makes them a relevant issue also for pathologists. In fact, the occurrence of such fatal events may represent a potential source of malpractice claims for cardiologists and it is noteworthy that AMI, in some manner, remains one of the most challenging areas with an associated high risk of alleged medical malpractice [158] and one of the clinical settings in which claims are most likely to arise [159, 160].

There is no doubt that prompt mechanical or/and pharmacological myocardial reperfusion represents the only realistic strategy in STEMI and that it has greatly improved AMI outcome. However, patients may have increasing optimistic expectations about the benefits of the procedures, as well as in many other cardiological clinical settings [161–163], and especially in cases with fatal outcome litigations and malpractice claims may arise thus leading to medicolegal autopsies which are critical in proving or excluding medical malpractice. It is now recognized that there are a spectrum of responses of the myocardium to ischemia/reperfusion [16, 157], and knowledge on the biochemical and molecular substrates of myocardial IRI has considerably improved. Reperfusion induces typical patterns of myocardial injury; contraction bands, calcium loading in the irreversibly injured myocytes, and hemorrhage in the region due to leakage of blood out of damaged blood vessels have been associated with IRI [16]. There is a growing appreciation that the pathobiologic response to ischemia/reperfusion injury is characterized by changes involving, among others, oxidative stress, mitochondria, and Ca^+^ homeostasis disturbance, with each leading to unique histomorphological footprint. As the cellular and molecular processes of myocardial IRI are more and more unravelled, the histopathology of reperfused AMI has been revisited and deserves further studies [3, 164]. We believe that, in postmortem examination in cases of fatal outcome of reperfused AMI, forensic experts should be very careful as this type of postmortem examination requires a deep knowledge and investigation of the complex ionic and biochemical alterations which could result in an unstable electrical substrate capable of initiating and sustaining arrhythmias. A sound knowledge of the pathophysiological changes underlying myocardial IRI and, namely, reperfusion arrhythmias is critical for forensic pathologists to make correct opinions concerning the real mechanism of death. Forensic pathologists, like clinicians, must think correlatively and move towards the explanation of the death on the basis of the underlying complex mechanisms. As a general concept, but mostly when deep pathophysiological derangements occur potentially leading to death, structural and anatomical knowledge obtained from autoptic observation is not so useful and cannot provide satisfactory explanations, independently of functional knowledge.

## 6. Therapeutic Challenges

Although there is no doubt that in AMI the reopening, as soon as possible, of occluded coronary arteries using either thrombolytic therapy or primary percutaneous coronary intervention is of vital importance for limiting the infarct size, thus representing an effective tool in AMI [162, 163], currently no similarly valid options exist in the treatment of myocardial IRI. Since the 1980s research has been focused on therapeutic agents that would render myocardial cells more resistant to the deleterious effects of ischemia and reperfusion [3, 164], the concept of “cardioprotection” encompasses the manipulation of the cellular events by different therapeutic tools during ischemia and reperfusion to reduce the amount of myocardial cells death [3, 164]. Ischemic conditioning strategies (ischemic preconditioning, IPC; remote ischemic preconditioning, RIPC; and ischemic postconditioning, iPOST) have been widely investigated in laboratory settings. Nevertheless, there still exists some difficulty in translating experimental results and controlled animal models into a heterogeneous population of human patients [12, 13, 24, 165]. An incomplete understanding of how cardioprotective signalling may be initiated at the level of the cardiomyocytes may, in part, explain the lack of success [166]. A deeper knowledge of the cellular and molecular mechanisms underlying IRI has led to the development of cardioprotective strategies, focusing on epigenetic regulation, limitation of cell death (both necrosis and apoptosis), stem cell regenerative therapies, gene therapy, and the use of growth factors [167]. Among the mechanisms through which postischemic myocardial damage has been shown to occur, mitochondrial dysfunction and the opening of MPTP are key steps. This crucial role renders them very attractive targets for therapeutic intervention [168–170]. In this context, a potential cardioprotective effect of intracoronary administration of 4-chlorodiazepam (4-CLD, a benzodiazepine derivative of diazepam) in animal models of IRI, has been recently demonstrated [171], thus suggesting a therapeutic role of intracoronary infusion of 4-CLD in AMI [171].

Furthermore, several lines of evidence support a potential role of platelet-rich plasma (PRP), an autologous product rich in growth factors obtained from a blood sample, in the healing of MI injury [172–175]. Platelets contain a wide amount of growth factors that are crucial in the reparative process following ischemic myocardial injury. In addition, they are rich in Factor XIII, a plasma transglutaminase, that has been shown to be critical in post-MI healing [176–178]. Factor XIII influences several steps of the reparative process, the formation of the three-dimensional fibrin meshwork, and the ECM components. Furthermore it is essential in adult stem cells recruitment, neoangiogenesis, and collagen deposit, thus playing a pivotal role at the intersection of several pathways involved in myocardial healing [179, 180].

## 7. Conclusions

AMI is a major cause of mortality worldwide. Early and successful myocardial reperfusion with either thrombolytic agents or primary percutaneous coronary intervention is the most effective strategy to reduce infarct size and improve clinical outcome. However, the process of restoring blood flow to the ischemic myocardium can induce myocardial reperfusion injury, which can paradoxically reduce the beneficial effects of myocardial reperfusion. Thus reperfusion itself may lead to accelerated and additional myocardial injury beyond that generated by ischemia alone [181]. Different clinical manifestations of this injury exist [13]; however, RAs remain the most important causes of sudden death following reperfusion therapy [182] even when the latter is technically successful. Thus myocardial IRI is both a critical clinical and medicolegal problem. For clinicians a better understanding of the pathophysiology of myocardial IRI may open the way to new therapeutic strategies [25, 182, 183]. For forensic pathologists, the value of fostering a knowledge of IRI pathophysiology should be highlighted as this can lead to an increased awareness of this potentially fatal event related to myocardial IRI, even in the case of optimal and early treatment. The clear investigation and comprehension of IRI may be an additional value which may diminish the risk of exposure of physicians to malpractice claims.
